# 2520. A Phase 1b Study of the Safety, Pharmacokinetics, and *Ex Vivo* Tick Killing of the Lyme Prophylaxis Candidate TP-05

**DOI:** 10.1093/ofid/ofad500.2138

**Published:** 2023-11-27

**Authors:** Jeremy J Lim, Christopher S Crean, Hilary A Partin, Jose M Trevejo, Seshadri Neervannan

**Affiliations:** Tarsus Pharmaceuticals, Inc., Irvine, California; Xyzagen, Inc., Pittsboro, North Carolina; Allegro Biopharma Associates, Raleigh, North Carolina; Tarsus Pharmaceuticals, Inc., Irvine, California; Tarsus Pharmaceuticals, Inc., Irvine, California

## Abstract

**Background:**

In the U.S., Lyme disease is the most common vector-borne disease caused by infection with *B. burgdorferi* bacteria following a bite by a tick vector. TP-05 (lotilaner oral capsule), an isoxazoline, is a selective inhibitor of insect and acarine GABA mediated chloride channels, which paralyzes the nervous system and eventually leads to death of ticks and other ectoparasites. This Phase 1b study was conducted to evaluate the safety, tolerability, food-effect, and pharmacokinetics of TP-05 after single and multiple dose administration in healthy subjects. In addition, serum samples from TP-05 dosed subjects were tested in an *ex vivo* tick kill model.

**Methods:**

40 healthy adult subjects were randomized across five single-ascending dose (SAD) cohorts to receive TP-05 or placebo (25, 100, 400, 600 mg under fed conditions, and 600 mg under fasted conditions). Subjects were followed up to Day 151. 24 healthy adult subjects were randomized across three multiple-ascending dose (MAD) cohorts to receive TP-05 or placebo under fed conditions (250 mg Day 1 and 75 mg Day 29; 250 mg Day 1 and 250 mg Day 29; 300 mg Day 1 and 75 mg weekly for 4 weeks). Subjects were followed through Day 151. Whole blood samples from the MAD cohorts were collected to evaluate the *ex vivo* lotilaner concentration dependent killing of ticks.

**Results:**

61 subjects (95.3%) completed the study. The most common study drug related treatment-emergent adverse event (TEAE) was headache (6 subjects [9.4%] overall; 5/6 receiving TP-05 and 1/6 receiving placebo) and it was not dose dependent. One placebo subject experienced a nonfatal serious adverse event (gunshot wound) and one subject discontinued the study due to COVID-19; both events were considered unrelated to study drug. TP-05 demonstrated a dose proportional increase in AUCs and C_max_ under fed conditions. T_max_ under fed conditions ranged from 5-8 hours post-dose. The half-life was approximately 8 weeks. *Ex vivo* tick feeding model showed killing of adult and nymph ticks as early as 2 hours post-dose. At Day 151, there was decreased survival indicating a sustained effect of TP-05.

**Conclusion:**

TP-05 was generally safe and well tolerated among healthy adult subjects. Data from this study supports TP-05’s mechanism of action and potential as a prophylactic therapy for Lyme disease.

**Disclosures:**

**Jeremy J. Lim, PharmD**, Roche Pharmaceuticals: Stocks/Bonds **Seshadri Neervannan, PhD**, Abbvie: Stocks/Bonds

